# The Ankyrin Repeat Domain Controls Presynaptic Localization of Drosophila Ankyrin2 and Is Essential for Synaptic Stability

**DOI:** 10.3389/fcell.2019.00148

**Published:** 2019-08-14

**Authors:** Tobias Weber, Raiko Stephan, Eliza Moreno, Jan Pielage

**Affiliations:** ^1^Department of Zoology and Neurobiology, University of Kaiserslautern, Kaiserslautern, Germany; ^2^Friedrich Miescher Institute for Biomedical Research, Basel, Switzerland

**Keywords:** Ankyrin, Ankryin repeat domain, synapse stability, cell adhesion molecules, microtubules, Drosophila, neurodegeneration

## Abstract

The structural integrity of synaptic connections critically depends on the interaction between synaptic cell adhesion molecules (CAMs) and the underlying actin and microtubule cytoskeleton. This interaction is mediated by giant Ankyrins, that act as specialized adaptors to establish and maintain axonal and synaptic compartments. In Drosophila, two giant isoforms of Ankyrin2 (Ank2) control synapse stability and organization at the larval neuromuscular junction (NMJ). Both Ank2-L and Ank2-XL are highly abundant in motoneuron axons and within the presynaptic terminal, where they control synaptic CAMs distribution and organization of microtubules. Here, we address the role of the conserved N-terminal ankyrin repeat domain (ARD) for subcellular localization and function of these giant Ankyrins *in vivo*. We used a P[acman] based rescue approach to generate deletions of ARD subdomains, that contain putative binding sites of interacting transmembrane proteins. We show that specific subdomains control synaptic but not axonal localization of Ank2-L. These domains contain binding sites to L1-family member CAMs, and we demonstrate that these regions are necessary for the organization of synaptic CAMs and for the control of synaptic stability. In contrast, presynaptic Ank2-XL localization only partially depends on the ARD but strictly requires the presynaptic presence of Ank2-L demonstrating a critical co-dependence of the two isoforms at the NMJ. Ank2-XL dependent control of microtubule organization correlates with presynaptic abundance of the protein and is thus only partially affected by ARD deletions. Together, our data provides novel insights into the synaptic targeting of giant Ankyrins with relevance for the control of synaptic plasticity and maintenance.

## Introduction

Structural integrity of cellular compartments depends on the coordinated control of cell-cell interactions mediated by cell adhesion molecules (CAMs) and the underlying cytoskeleton. The interplay between cell surface molecules and the actin and microtubule cytoskeleton is particularly evident during the development and structural plasticity of synaptic connections in the nervous system (Giagtzoglou et al., 2009; Goellner and Aberle, 2012; Yogev and Shen, 2014). Precise control of microtubule dynamics shapes axonal growth and the establishment of synaptic and dendritic domains (Conde and Cáceres, 2009; Rao and Baas, 2018). The positioning and stability of CAMs at the plasma membrane is coordinated by intracellular linker molecules like Ankyrins that provide an association to the cellular cytoskeleton (Bennett and Lorenzo, 2016). Ankyrins are multi-domain proteins that bind to transmembrane proteins and the spectrin skeleton to form and organize subcellular domains (Bennett and Lorenzo, 2016). In the nervous system, giant Ankyrins organize and establish axonal domains and are essential for the maintenance of synaptic structures (Koch et al., 2008; Pielage et al., 2008; Galiano et al., 2012; Ho et al., 2014; Smith et al., 2014; Zhang and Rasband, 2016). Like all Ankyrins, giant Ankyrins are composed of an N-terminal ankyrin repeat domain (ARD), a spectrin-binding domain, and a death domain. In addition, they include large C-terminal domains that were acquired through the incorporation of large exons (Bennett and Lorenzo, 2016). While these additional domains are not conserved between invertebrate and vertebrate giant Ankyrins (Bennett and Walder, 2015), the giant Ankyrins share common functional roles in the axonal organization of microtubules (Pielage et al., 2008; Jenkins et al., 2015; Stephan et al., 2015). Structurally, the ARD consists of 24 conserved ankyrin helix-turn-helix repeats that are each 33 amino acid (aa) long. Together, these repeats form a superhelical solenoid with an elongated conserved binding grove (Wang et al., 2014) that serves as a binding module for multiple, divergent transmembrane proteins including cell adhesion molecules (Davis and Bennett, 1994; Zhang et al., 1998; Kizhatil et al., 2007; Alpizar et al., 2019) and voltage gated sodium and Kv3.1 channels (Srinivasan et al., 1988; Devaux et al., 2003; Lemaillet et al., 2003; Xu et al., 2007). All giant Ankyrins bind CAMs of the L1 family (Chen et al., 2001; Hortsch et al., 2002; Godenschwege et al., 2006; Enneking et al., 2013; Wang et al., 2014; Siegenthaler et al., 2015) and this interaction is essential for the localization of giant Ankyrins to the AIS (Wang et al., 2014). In contrast, the binding to voltage-gated sodium channels only appeared within the chordate lineage (Hill et al., 2008). These differential binding properties are reflected by both shared and divergent organizational roles of giant Ankyrins within vertebrate and invertebrate neurons. In vertebrate neurons giant Ankyrins are essential for the clustering and maintenance of voltage-gated channels at the axon initial segment and the nodes of Ranvier (Zhang and Rasband, 2016). In addition, in both vertebrate and invertebrate neurons, giant Ankyrins contribute to the organization of axonal and synaptic protein domains and synaptic stability (Chen et al., 2001; Pielage et al., 2008; Enneking et al., 2013; Smith et al., 2014; Stephan et al., 2015). While targeting and organization of the AIS and nodes of Ranvier by giant Ankyrins is relatively well understood (Zhang and Rasband, 2016) we currently have only limited knowledge regarding the molecular mechanisms mediating the localization and organizational roles of giant Ankyrins at pre- and postsynaptic compartments *in vivo*.

In this study, we use the Drosophila *ankyrin2* (*ank2*) gene to determine the importance of the N-terminal ARD for synaptic targeting and function of giant Ankyrins. Drosophila *ank2* encodes two major giant isoforms, Ank2-L (large, 450 kDa) and Ank2-XL (extra-large, 1200 kDa) that are selectively expressed in the nervous system (Koch et al., 2008; Pielage et al., 2008; Stephan et al., 2015). In motoneurons, both isoforms are highly abundant in the axon and at the presynaptic nerve terminal of the neuromuscular junction (NMJ). Ank2-L provides a link between the synaptic CAM Neuroglian (Nrg, L1CAM homolog) and microtubules to promote synaptic stability (Koch et al., 2008; Pielage et al., 2008; Enneking et al., 2013; Stephan et al., 2015). Ank2-L also controls the presynaptic localization of Ank2-XL that, in turn, controls the organization of presynaptic microtubules in association with the microtubule-associated protein Futsch (MAP1B homolog) (Stephan et al., 2015). By utilizing Drosophila *in vivo* genetics, we address the importance of individual repeat units within the ARD for the localization, maintenance and function of giant Ankyrin2 proteins at the synapse.

## Results

### Ank2-L Controls the Synaptic Localization of Ank2-XL

**FIGURE 1:**
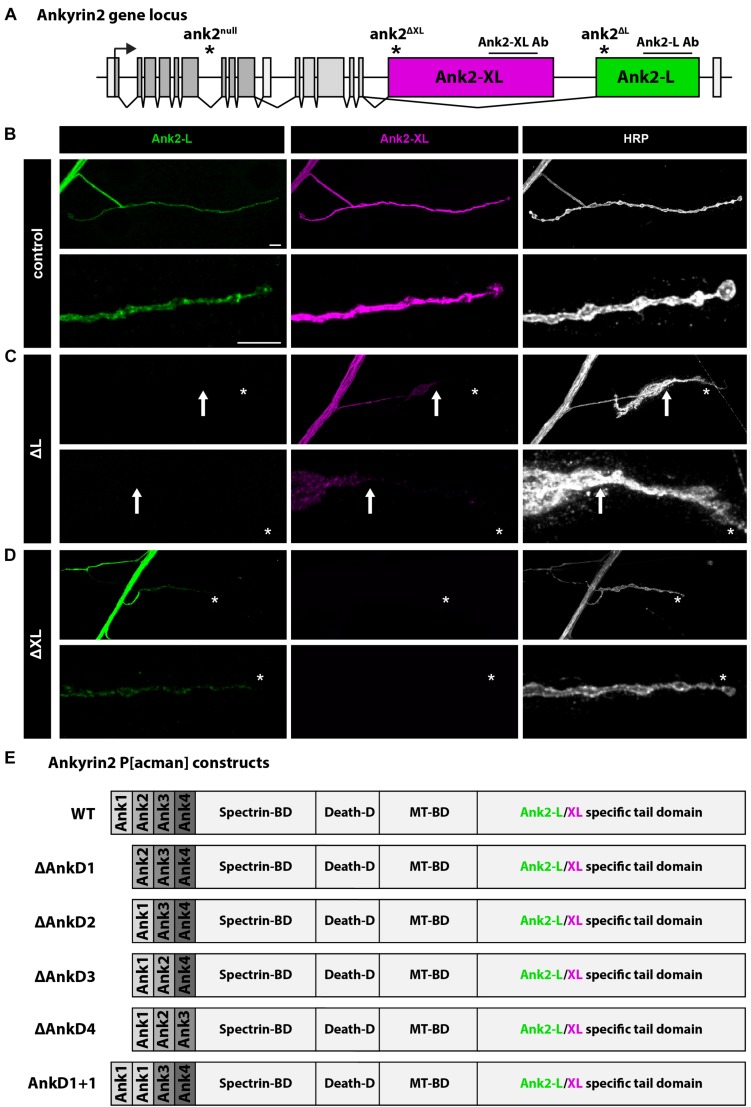
Analysis of Ank2-L and Ank2-XL. **(A)** Schematic of the Drosophila *ank2* genomic locus. The position of the *ank2-L* and *ank2-XL* specific exons and the positions of the specific mutations and antibody epitopes are indicated. **(B)** Analysis of Ank2-L and Ank2-XL distribution in control animals. Both proteins are present in all synaptic regions demarcated by the neuronal membrane marker HRP. **(C)** In *ank2^ΔL^* (ΔL) mutant animals Ank2-L is completely absent and a severe perturbation of synaptic Ank2-XL distribution and levels can be observed. Ank2-XL is no longer present in distal boutons (arrow, asterisk). **(D)** In *ank2^ΔXL^* (ΔXL) mutant animals Ank2-XL is absent. Ank2-L levels are reduced but the presynaptic distribution is not perturbed (asterisk). **(E)** Schematic overview of the P[acman] constructs used to analyze the contribution of different parts of the ARD for Ank2-L and Ank2-XL localization and function. Each AnkD region includes six ankyrin repeats. Scale bars in **(B)** apply to **(B–D)** and represent 10 μm.

The Drosophila *ank2* gene encodes for the two alternative giant isoforms, Ank2-L and Ank2-XL, that are characterized by unique C-terminal tail domains (Figure 1A). In larval motoneurons Ank2-L and Ank2-XL are highly abundant in axons and within the presynaptic terminal of the NMJ (Figure 1B; Koch et al., 2008; Pielage et al., 2008; Stephan et al., 2015). Using isoform specific mutations, it has been previously demonstrated that Ank2-L is essential for synapse stability and controls the synaptic localization of Ank2-XL that in turn contributes to presynaptic bouton organization via interactions with Futsch and the microtubule cytoskeleton (Stephan et al., 2015). Consistently, we observed that loss-of-function mutations of *ank2-L* lack any Ank2-L protein and severely impaired the presynaptic levels and distribution of Ank2-XL (Figure 1C). In contrast, mutations in *ank2-XL* abolished Ank2-XL staining but only slightly reduced Ank2-L levels without affecting presynaptic distribution (Figure 1D). To determine the contribution of the N-terminal ARD for Ank2-L and Ank2-XL localization and function we generated P[acman]-based deletion constructs that enable the generation of genomic rescue constructs mimicking knock-in like mutations (Venken et al., 2006; Siegenthaler et al., 2015; Stephan et al., 2015). To gain insights into the role of different parts of the ARD that forms a large superhelical solenoid (Wang et al., 2014) we subdivided the 24 ankyrin repeat domain into four subunits each containing six ankyrin repeats (here referred to as AnkD1–4). This subdivision is based on earlier studies characterizing putative Ankyrin-binding partners and takes into consideration the observation that 4–5 ankyrin repeats form a folded structural unit (Bennett and Baines, 2001; Li et al., 2006; Wang et al., 2014). To control for potential disruptive effects of deleting parts of the ARD super-structure we also generated a replacement construct in which we exchanged sequences of domain 2 (repeats 7–12) with sequences of domain 1 (repeats 1–6) (Figure 1E). In combination with the isoform-specific *ank2* mutations these rescue constructs allow us to determine the importance of individual parts of the ARD for the localization and function of Ank2-L and Ank2-XL. The presence of the wild type *ank2* P[acman] construct completely rescued viability of the *ank2* null mutant animals that otherwise die as early first instar larvae (Pielage et al., 2008; Stephan et al., 2015). In contrast, introduction of ARD deletions significantly reduced the rescue ability. While AnkD2, 3 or the replacement of AnkD2 with AnkD1 (AnkD1+1) failed to restore viability, AnkD1 rescued viability to adult stages and AnkD4 to pupal stages. These results indicate differential functional requirements of the ARD subdomains for animal survival. Importantly, combinations of the P[acman] constructs with isoform specific mutations that survive to larval stages (Koch et al., 2008; Pielage et al., 2008; Stephan et al., 2015) enabled us to determine the selective requirements of the ARD subdomains for Ank2-L and Ank2-XL localization and function.

### Presynaptic Localization of Ank2-L Depends on AnkD2, 3, and 4

**FIGURE 2:**
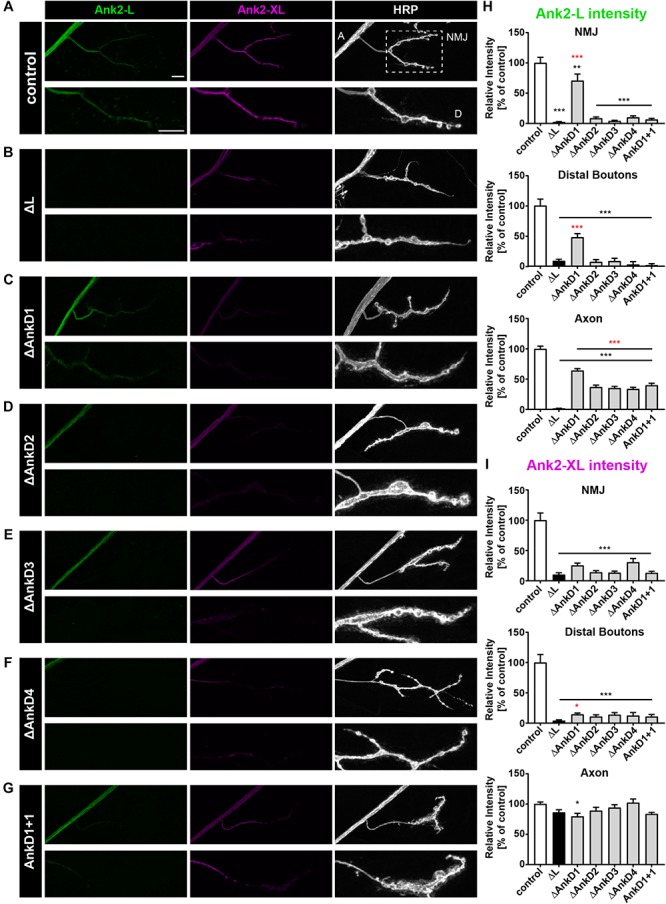
Analysis of ARD-dependent localization of Ank2-L and Ank2-XL in *ank2^ΔL^* mutants. **(A–G)** Analysis of Ank2-L (green) and Ank2-XL (magenta) localization at muscle 4 NMJs (HRP, white). **(A)** In control animals (*P[ank2_wt]*/+; *ank2^ΔL^*/*ank2*^null^) presence of the wild type P[acman] *ank2* constructs completely restores NMJ localization of Ank2-L and Ank2-XL. Areas for quantification of intensities are indicated in the HRP channel (A, axon, D, distal bouton, NMJ). **(B)** In *ank2^ΔL^/ank2^null^* mutant animals Ank2-L is absent and levels of Ank2-XL are severely reduced at the NMJ but not in the axon. **(C–F)** Analysis of the four AnkD deletion mutants (*P[ank2_ΔAnkDx]*/+; *ank2^ΔL^*/*ank2*^null^) reveals severe perturbations of Ank2-L level at the NMJ. These effects are less severe for the *AnkD1* mutation. All deletion mutations significantly reduce Ank2-XL distribution at the NMJ. **(G)** Analysis of the AnkD1+1 construct in which the second AnkD has been replaced by the first AnkD. Both Ank2-L and Ank2-XL distribution are perturbed comparable to the deletion of the second AnkD. **(H)** Quantification of Ank2-L level at the NMJ, within distal boutons and in the axon (*n* = 11–12 muscle 4 NMJs, three animals/genotype). **(I)** Quantification of Ank2-XL level at the NMJ, in distal boutons and in the axon (*n* = 11–12 muscle 4 NMJs, three animals/genotype). Scale bars in **(A)** apply to **(A–G)** and represent 10 μm. Error bars indicate SEM; ^*^p < 0.05, ^∗∗^*p* < 0.01, ^∗∗∗^*p* < 0.001 (ANOVA); black asterisks represent comparisons to controls; red asterisks represent comparisons to *ank2^ΔL^* mutants.

First, we determined how deletion of individual AnkDs affected the localization of the different Ank2 isoforms in the background of the *ank2-L* mutation. We quantified relative fluorescence intensity levels in the axon (A), the presynaptic NMJ and in distal boutons (D) in third instar larvae (Figure 2A, areas highlighted) and compared these values to animals expressing a wild-type version of the *ank2* P[acman] construct in the *ank2^ΔL^* mutant background (control = *P[ank2_wt]*/+; *ank2^ΔL^/ank2^null^*) (Figure 1A). The presence of the wildtype *ank2* P[acman] construct completely restored Ank2-L and Ank2-XL localization in motoneurons and restored all synaptic phenotypes associated with the loss of *ank2* (this study, Stephan et al., 2015). In contrast, rescue constructs lacking AnkD2, 3, or 4 or replacement of AnkD2 with AnkD1 (AnkD1+1) did not significantly restore protein levels, and we observed an almost complete absence of Ank2-L at the NMJ (Figures 2B–H, Supplementary Figure S1, and Supplementary Table S1). Importantly, Ank2-L protein levels within axonal regions were rescued to approximately 30% wild type intensity demonstrating that all P[acman] rescue constructs encode functional proteins. These severe effects on presynaptic Ank2-L localization were likely not due to general structural defects as the deletion construct lacking the first AnkD provided significant rescue of Ank2-L localization at the NMJ including proximal and distal bouton regions (Figures 2C,H and Supplementary Figure S1). We next analyzed the distribution of Ank2-XL in these animals. In *ank2^ΔL^* mutant animals we observed a severe reduction in Ank2-XL levels at the NMJ but not in axons consistent with our prior report (Figures 2B,I; Stephan et al., 2015). Interestingly, deletion or manipulation of any AnkD led to a significant loss of Ank2-XL at the NMJ [Figures 2C–G (middle panels),I and Supplementary Figure S1]. This includes the Δ*AnkD1* construct that provided partial rescue of Ank2-L levels at the NMJ (Figures 2C,I). In contrast, protein abundance within axonal regions was not impaired [Figures 2B–G(middle panels),I and Supplementary Figure S1]. These results show that impaired Ank2-L localization due to deletions of individual ARD regions affect not only presynaptic Ank2-L but also Ank2-XL localization.

**FIGURE 3:**
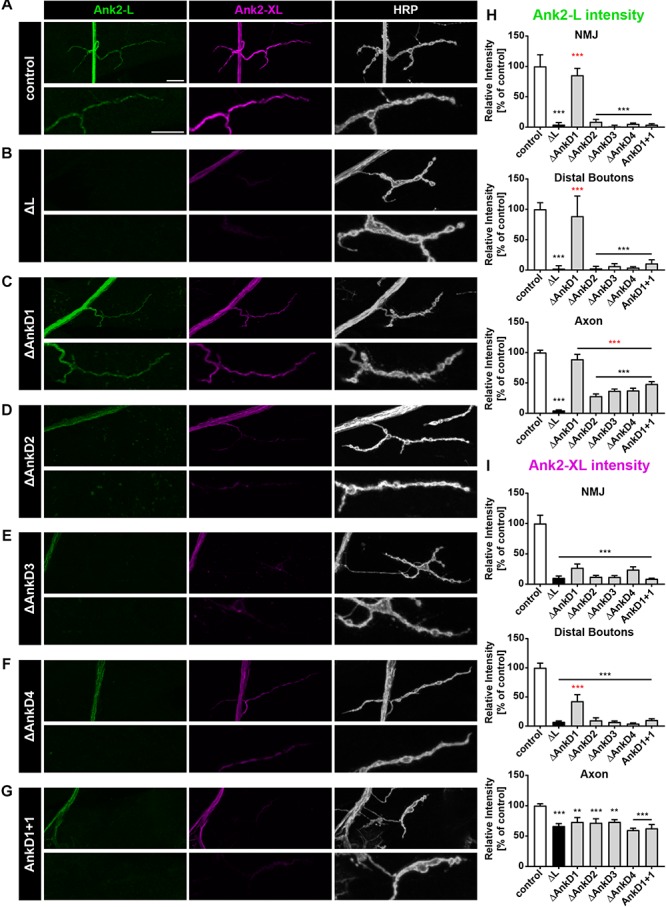
Analysis of ARD-dependent localization of Ank2-L and Ank2-XL in second instar *ank2^ΔL^* mutants. **(A–G)** Analysis of Ank2-L (green) and Ank2-XL (magenta) localization at muscle 4 NMJs (HRP, white). **(A)** In control animals (*P[ank2_wt]*/+; *ank2^ΔL^/ank2^null^*) Ank2-L and Ank2-XL level are restored compared to *ank2^ΔL^/ank2^null^* mutants. **(B)** In *ank2^ΔL^/ank2^null^* mutant animals Ank2-L is absent and levels of Ank2-XL are severely reduced at the NMJ and also in the axon. **(C–G)** Analysis of the AnkD manipulations (*P[ank2_ΔAnkDx]*/+; *ank2^ΔL^/ank2^null^*) reveals alterations of Ank2-L and Ank2-XL similar to those in third instar mutant larvae. The Δ*AnkD1* rescue construct almost completely restores Ank2-L level. All deletion mutations significantly reduce Ank2-XL distribution at the NMJ and in axons. **(H)** Quantification of Ank2-L level at the NMJ, within distal boutons and in the axon (*n* = 11–12 muscle 4 NMJs, three animals/genotype). **(I)** Quantification of Ank2-XL level at the NMJ, in distal boutons and in the axon (*n* = 11–12 muscle 4 NMJs, three animals/genotype). Scale bars in **(A)** apply to **(A–G)** and represent 10 μm. Error bars indicate SEM; ^∗∗^*p* < 0.01, ^∗∗∗^*p* < 0.001 (ANOVA); black asterisks represent comparison to controls; red asterisks represent comparisons to *ank2^ΔL^* mutants.

To determine whether these effects were due to defects in the initial localization or maintenance of the protein we next analyzed protein distribution in second instar larvae, when defects in synaptic stability or organization are much less pronounced (Stephan et al., 2015). At these earlier developmental stage, we observed largely identical results with three important exceptions: First, the Δ*AnkD1* construct now provided a complete rescue of Ank2-L protein levels despite the fact that none of the other rescue constructs restored Ank2-L distribution at the NMJ (Figures 3A–H and Supplementary Figure S1). Second, in contrast to the third instar larvae, the levels of Ank2-XL in the axons are slightly but significantly compromised at this earlier developmental stage (Figures 3A–G,I and Supplementary Figure S1). And third, the Δ*AnkD1* mutation provides some rescue of Ank2-XL level at distal boutons indicating that Ank2-XL targeting depends on appropriate localization of Ank2-L (Figures 3C,I).

Together these results demonstrate a critical requirement of AnkD2-4 for presynaptic localization of Ank2-L. Ank2-XL localization in turn depends on appropriate targeting of Ank2-L and the presence of the entire ARD of Ank2-L.

### Ankyrin Repeats Contribute to Synaptic Localization of Ank2-XL

**FIGURE 4:**
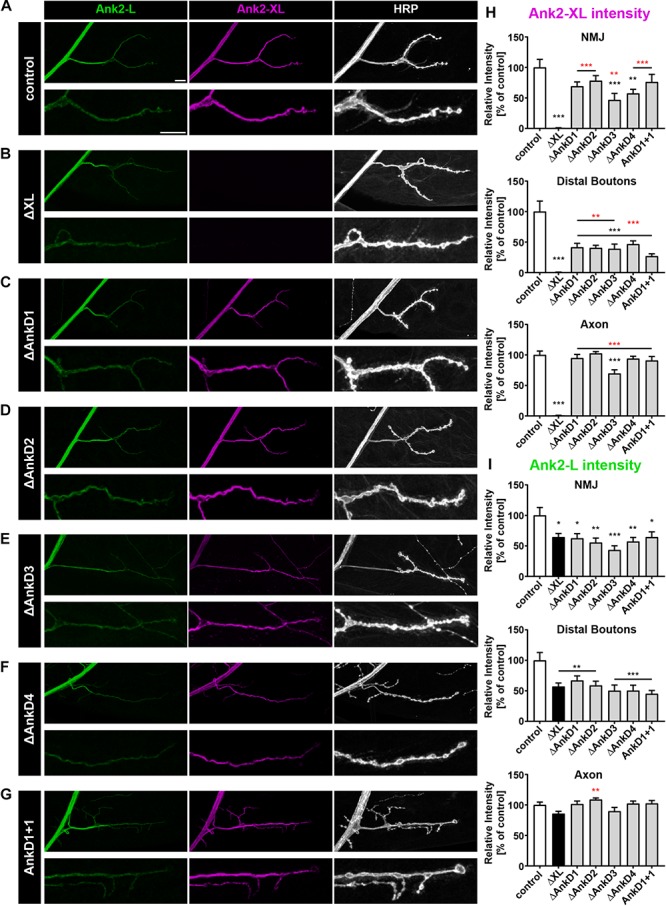
Analysis of ARD-dependent localization of Ank2-L and Ank2-XL in *ank2^ΔXL^* mutants. **(A–G)** Analysis of Ank2-L (green) and Ank2-XL (magenta) localization at muscle 4 NMJs (HRP, white). **(A)** In control animals (*P[ank2_wt]*/+; *ank2^ΔXL^/ank2^ΔXL^*) levels of Ank2-L and Ank2-XL are restored compared to *ank2^ΔXL^* mutants. **(B)** In *ank2^ΔXL^/ank2^ΔXL^* mutant animals Ank2-XL is absent and levels of Ank2-L are slightly reduced at the NMJ but not in the axon. **(C–G)** Analysis of the AnkD manipulations (*P[ank2_ΔAnkDx]*/+; *ank2^ΔXL^/ank2^ΔXL^*) reveals reductions of Ank2-L and Ank2-XL level at the NMJ. Ank2-XL levels are significantly reduced in the axon and NMJ when the third AnkD was deleted. **(H)** Quantification of Ank2-XL level at the NMJ, within distal boutons and in the axon (*n* = 12 muscle 4 NMJs, three animals/genotype). **(I)** Quantification of Ank2-L level at the NMJ, in distal boutons and in the axon (*n* = 12 muscle 4 NMJs, three animals/genotype). Scale bars in **(A)** apply to **(A–G)** and represent 10 μm. Error bars indicate SEM; ^*^p < 0.05, ^∗∗^*p* < 0.01, ^∗∗∗^*p* < 0.001 (ANOVA); black asterisks represent comparison to controls; red asterisks represent comparisons to *ank2^ΔXL^* mutants.

We next determined the importance of the AnkDs for synaptic targeting of Ank2-XL by analyzing the P[acman] rescue constructs in the *ank2^ΔXL^* mutant background. In contrast to Ank2-L, the individual AnkDs are less essential for synaptic targeting of Ank2-XL. For all AnkD deletions we observed a significant rescue of Ank2-XL localization at the NMJ compared to the *ank2^ΔXL^* mutation (Figures 4A–H). However, deletion of the third and fourth AnkDs significantly reduced protein levels at the NMJ and all constructs showed a significant reduction of protein levels at distal synaptic boutons (Figures 4A–H). The third AnkD was most important for Ank2-XL localization as this was the only deletion leading to a significant reduction of axonal protein levels (Figures 4E,H). Importantly, the complete rescue of axonal Ank2-XL localization by all constructs with the exception of Δ*AnkD3* validated that all observed alterations in synaptic localization were due to specific ARD-effects and not to general perturbations of protein expression. Our detailed quantification analysis also demonstrated a requirement of Ank2-XL for the synaptic localization of Ank2-L (Figures 4B,I). This Ank2-XL dependent localization of Ank2-L required the presence of the entire ARD. All *AnkD* deletion mutations and the *ank1+1* construct failed to restore Ank2-L levels at the NMJ with Δ*AnkD3* having the strongest impact (Figures 4A–G,I and Supplementary Figure S1). These results demonstrate unique requirements of individual AnkDs for the localization of Ank2-L and Ank2-XL and reveal a striking co-dependency between the two giant Ank2 isoforms.

### AnkDs Are Essential for Ank2-L Mediated Synaptic Stability

**FIGURE 5:**
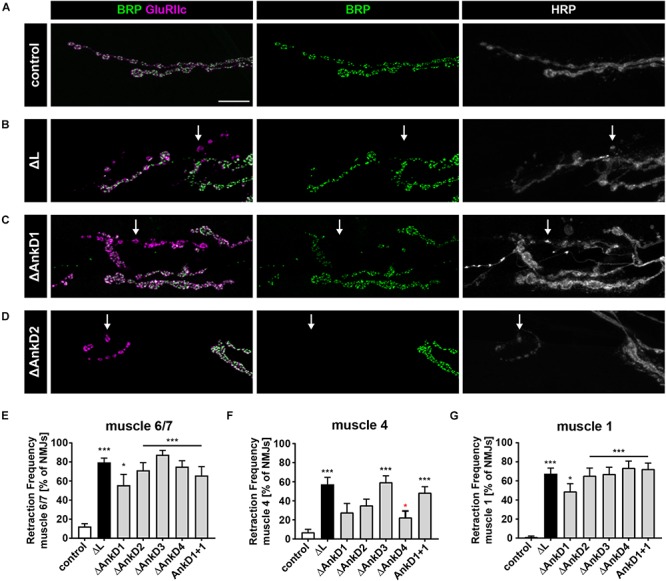
ARD is essential for Ank2-L mediated synaptic stability. **(A–D)** Analysis of synaptic stability using the presynaptic active zone marker Brp (green), the postsynaptic marker GluRIIc (magenta) and the presynaptic membrane marker HRP (white). **(A)** In control animals (*P[ank2_wt]*/+; *ank2^ΔL^/ank2^null^*) presence of the wild type P[acman] *ank2* constructs completely restores the synaptic stability defects associated with the *ank2^ΔL^* mutation. **(B)** In the absence of Ank2-L synaptic retractions are evident as postsynaptic GluRIIc clusters no longer opposed by the presynaptic maker Brp (arrow). The presynaptic membrane is fragmented in these areas. **(C,D)** Rescue constructs containing deletions of the first **(C)** and second **(D)** AnkD fail to completely restore synaptic stability defects in the *ank2^ΔL^* mutant background (arrows). **(E–G)** Quantification of the frequency of synaptic retractions on muscles 6/7 **(E)**, muscle 4 **(F)** and muscle 1 **(G)** in control, *ank2^ΔL^* and Δ*AnkD* mutants (*n* = 72–111 muscle 6/7 NMJs, 12–19 animals; *n* = 66–101 muscle 4 NMJs, 11–19 animals; *n* = 71–104 muscle 1 NMJs; 12–19 animals). Scale bars in **(A)** apply to **(A–D)** and represent 10 μm. Error bars indicate SEM; ^*^p < 0.05, ^∗∗∗^*p* < 0.001 (ANOVA); black asterisks represent comparisons to controls; red asterisks represent comparisons to *ank2^ΔL^* mutants.

To determine to which extent the AnkD deletions impact the functional requirements of Ank2-L and Ank2-XL we next analyzed synaptic stability. In wild type animals, the Drosophila NMJ represents a very stable structure that displays few synaptic retractions and that is characterized by the perfect apposition of the presynaptic marker Bruchpilot (Brp) and postsynaptic glutamate receptor clusters (GluR) (Figure 5A) (Pielage et al., 2005). In contrast, mutations in *ank2* and in the *ank2* associated genes *spectrin*, *adducin* (*hts*), or *Nrg* (L1CAM homolog) lead to severe impairments of synaptic maintenance (Koch et al., 2008; Pielage et al., 2005, 2008, 2011; Enneking et al., 2013; Stephan et al., 2015). While the presence of the wild type *ank2* P[acman] construct perfectly restored the synaptic stability defects associated with the *ank2^ΔL^* mutation (Figures 5A,B,E–G; see also Stephan et al., 2015), the absence or replacement of any AnkD region significantly impaired synaptic stability evident by GluR clusters no longer opposed by the presynaptic marker Brp (Figures 5C–G). While all AnkDs contributed to synapse maintenance, the analysis of three different muscle groups along the ventral-dorsal axis revealed clear differences between the importance of individual domains. Consistent with our observation that the AnkD1 mutation has the least impact on presynaptic Ank2-L localization we observed a partial rescue of the *ank2^ΔL^* phenotype on muscle 4 and equally reduced retraction rates and severities on muscles 6/7 and 1 (Figures 5C,E–G and Supplementary Figure S2A). Similarly, deletions of AnkD2 and 4 resulted in a partial rescue of synaptic stability on muscle 4 (Figures 5D,F and Supplementary Figure S2A). In contrast, deletion of domain 3 failed to restore synaptic maintenance. These results show that presynaptic Ank2-L abundance correlates, at least partially, with synapse stability and that individual AnkD contributing to a different extent to presynaptic maintenance. Consistent with prior reports (Stephan et al., 2015), we did not observe any impairment of synaptic stability in *ank2^ΔXL^* mutants or in the *AnkD* deletions in the *ank2^ΔXL^* background (Supplementary Figure S2B). Thus, despite a reduction in both Ank2-XL and Ank2-L protein in these animals the remaining levels are sufficient to support structural synaptic stability.

### Ank2-L Dependent Organization of Synaptic CAMs

**FIGURE 6:**
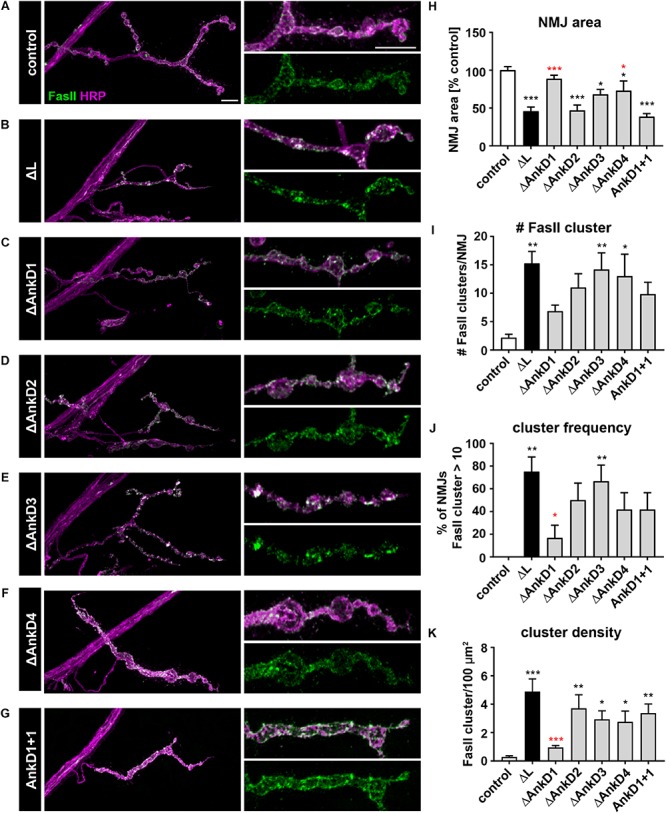
Analysis of ARD-dependent control of synaptic CAM organization. **(A–G)** Analysis of the synaptic distribution of the CAM Fas II (green) at type Ib muscle 4 NMJs marked by HRP (magenta). In contrast to control animals (*P[ank2_wt]*/+; *ank2^ΔL^/ank2^null^*) **(A)** absence of Ank2-L **(B)** or deletions/manipulations of the AnkDs **(C–G)** result in severe perturbations of the organization of the peri-active zone associated NCAM homolog Fas II. **(H–K)** Quantification of NMJ area **(H)**, number of Fas II cluster per NMJ **(I)**, frequency of NMJs displaying more than 10 Fas II cluster **(J)** and Fas II cluster density per 100 μm^2^ **(K)** (*n* = 12 muscle 4 NMJs, three animals). Scale bars in **(A)** apply to **(A–G)** and represent 10 μm. Error bars indicate SEM; ^*^p < 0.05, ^∗∗^*p* < 0.01, ^∗∗∗^*p* < 0.001 (ANOVA); black asterisks represent comparisons to controls; red asterisks represent comparisons to *ank2^ΔL^* mutants.

Since it has been shown that loss of Ank2-L results in aberrant accumulations of the CAMs Fasciclin II (Fas II, NCAM homolog) and Nrg (L1-CAM homolog, Pielage et al., 2008) we next analyzed the distribution of Fas II and Nrg in *AnkD* mutant animals. In control animals (*P[ank2_wt]*; *ank2^ΔL^/ank2^null^*) Fas II forms a honeycomb-like pattern in the peri-active zones of synaptic boutons of type Ib NMJs (Figure 6A). This pattern is severely perturbed in *ank2^ΔL^* mutants with Fas II accumulating in large clusters at type Ib NMJs (Figures 6B,I–K). In addition, the area of the NMJ was significantly reduced in these animals (Figures 6B,H). Quantification revealed the strongest Fas II clustering phenotype for Δ*AnkD3* mutants (Figures 6C–G,I,J) correlating with the synaptic retraction data for the individual AnkD deletions (Figure 5). Compared to *ank2^ΔL^* mutants we observed a partial improvement of the Fas II phenotype in Δ*AnkD1* and Δ*AnkD2* mutants (Figures 6C–G,I–K). However, as NMJ area is most severely affected in Δ*AnkD2* mutants the normalization of cluster density per NMJ showed a significant rescue of the phenotype only in Δ*AnkD1* mutants (Figures 6H,K). At type Is NMJs, we observed similar but less pronounced perturbations of Fas II distribution, probably due to the smaller size of individual boutons within these NMJs (Supplementary Figures S3A,B). Consistently, we detected almost identical disruptions of Nrg localization at both type Ib and type Is muscle 4 NMJs (Supplementary Figures S3C–F). Together, these data demonstrate that reduced synaptic levels of Ank2-L due to *AnkD* deletions partially correlate with impairments of synaptic CAM organization and increased perturbation of synaptic stability.

### Ank2-XL Dependent Control of Presynaptic Microtubule Organization

**FIGURE 7:**
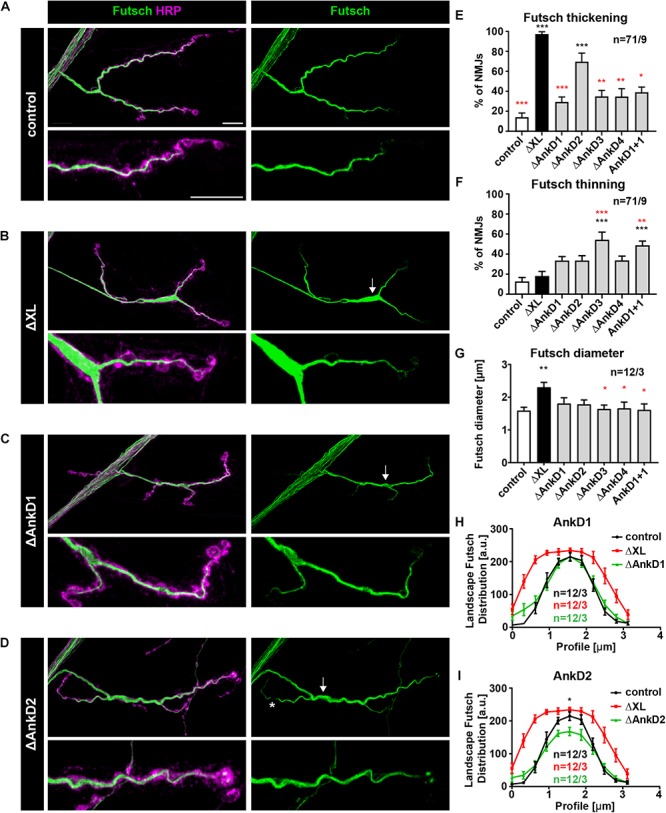
Analysis of ARD-dependent control of synaptic microtubule organization. **(A–D)** Analysis of the distribution of the microtubule marker Futsch/MAP1B (green) at muscle 4 NMJs. In control animals (*P[ank2_wt]*/+; *ank2^ΔXL^/ank2^ΔXL^*) Futsch marks the filamentous organization of presynaptic microtubules **(A)**. In the absence of Ank2-XL Futsch accumulates within synaptic boutons of central areas of the NMJ (thickening, arrow) **(B)**. Deletion mutations of the ARD cause only minor alterations of Futsch organization, thickenings (arrow) and thinnings (asterisks) **(C,D)**. **(E)** Quantification of aberrant thickenings of the Futsch staining (*n* = 71 muscle 4 NMJs, nine animals). **(F)** Quantification of aberrant thinnings of the Futsch staining (*n* = 71 muscle 4 NMJs, nine animals). **(G)** Quantification of the maximal Futsch diameter within NMJs (*n* = 12 muscle 4 NMJs, three animals). **(H,I)** Quantification of the Futsch profile within boutons of controls (black trace), *ank2^ΔXL^* (red trace), Δ*AnkD1* (green trace in **H**) and Δ*AnkD2* (green trace in **G**) mutants (*n* = 12 muscle 4 NMJs, three animals). Scale bars in **(A)** apply to **(A–D)** and represent 10 μm. Error bars indicate SEM; ^*^p < 0.05, ^∗∗^*p* < 0.01, ^∗∗∗^*p* < 0.001 (ANOVA); black asterisks represent comparisons to controls; red asterisks represent comparisons to *ank2^ΔXL^* mutants.

Finally, we tested the impact of the *AnkD* deletions on Ank2-XL dependent organization of the presynaptic microtubule cytoskeleton. Prior studies showed that Ank2-XL controls presynaptic microtubule organization via the microtubule-associated protein Futsch (MAP1B homolog) (Stephan et al., 2015). Consistent with these results we observed a severe disruption of Futsch organization in *ank2^ΔXL^* mutants that could be completely rescued in control animals (Figures 7A,B,E–G). In contrast, all *AnkD* deletions failed to completely restore Futsch organization (Figures 7C–F). In contrast to the complete absence of Ank2-XL aberrant increases in Futsch diameter (Figures 7B,E,G) were less frequent and less severe in the different ARD deletion mutations (Figures 7C–E,G). Interestingly, in these animals we also observed a significant increase in areas with small Futsch diameters (Figures 7D,F) The most severe Futsch thickening phenotype was observed for Δ*AnkD2* mutants (Figure 7E) but this phenotype was qualitatively different from the complete Ank2-XL loss-of-function phenotype as Futsch intensity was significantly reduced at central areas of the presynaptic terminal (Figures 7D,H,I). These results demonstrate that partial impairments of presynaptic Ank2-XL localization have only minor consequences for synaptic microtubule and NMJ organization.

## Discussion

Giant ankyrins serve as central organizing adaptor molecules in both invertebrate and vertebrate neurons. Here, we provide first insights into the control of synaptic localization and function of giant Ankyrins by the N-terminal ankyrin repeat domain *in vivo*. By selectively deleting subdomains of the ARD of *ank2* we unravel critical requirements of specific regions of the ARD for the synaptic localization of the neuronal specific giant isoforms Ank2-L and Ank2-XL. Our data demonstrate that the N-terminal domain controls not only synaptic targeting of individual isoforms but also contributes to synaptic localization of the alternative isoform. The functional requirements of Ank2-L and Ank2-XL for synaptic stability and microtubule organization clearly correlate with ARD-dependent regulation of protein abundance at the presynaptic terminal, with individual subdomains providing unique functional features (Supplementary Figure S4).

### Ankyrin Domains Control Presynaptic Localization of Ank2-L and Ank2-XL

The N-terminal ARD mediates membrane-association of Ankyrins and is essential for the subcellular localization and organization of transmembrane binding partners (Srinivasan et al., 1988; Davis and Bennett, 1994; Zhang et al., 1998; Devaux et al., 2003; Lemaillet et al., 2003; Kizhatil et al., 2007; Xu et al., 2007; Alpizar et al., 2019). Prior work largely focused on the organizational roles of giant Ankyrins at the AIS and nodes of Ranvier in vertebrate neurons. Our *in vivo* analysis of ARD deletions now revealed a critical importance of this domain for the localization of Ank2-L at the presynaptic terminal of motoneurons. The synaptic localization of Ank2-L depends on AnkD2–4 (repeats 7–24) while the first domain (repeats 1–6) only had a minor impact on protein abundance. Interestingly, axonal targeting of Ank2-L was only partially affected by these deletions. These results indicate that separate and distinctive mechanisms exist *in vivo* that enable selective localization of giant Ankyrins in axons and within the presynaptic motoneuron terminal. As we observed similar localization defects already at earlier stages of development it argues that AnkD2–4 are essential for initial synaptic localization. We previously demonstrated that a fusion protein comprising the specific C-terminal tail domain of Ank2-L efficiently localizes to both axonal and synaptic areas (Stephan et al., 2015). The demonstration that synaptic localization of full length Ank2-L requires an intact ARD indicates that subcellular distribution is precisely regulated and not achieved by passive distribution within the neuron. Of particular interest is the observation that the first six ankyrin repeats are dispensable for synaptic localization of Ank2-L in the *ank2^ΔL^* mutant background. The recent characterization of the structure of the human ARD demonstrated distinct and independent binding sites for voltage-gated sodium channels (Nav1.2) and for L1-family CAMs (Neurofascin) *in vitro* and in cultured neurons (Wang et al., 2014). Interestingly, in cultured hippocampal neurons the L1-family CAM binding sites within AnkD2 (ankyrin repeats 8–9 and 11–13) are essential for clustering of the 270 kDa AnkG isoform at the AIS. In contrast, AnkG lacking the Nav1.2 binding site within AnkD1 (ankyrin repeats 2–6) efficiently localized to the AIS but failed to cluster Nav channels at the AIS (Wang et al., 2014). Thus, similar to the situation at the vertebrate AIS, the synaptic localization of Drosophila Ank2-L largely depends on interactions with L1-family CAMs or alternative transmembrane proteins that occupy binding sites within the central and C-terminal part of the ARD. This localized L1-CAM-Ankyrin complex can then serve as an assembly platform for additional molecules like voltage-gated sodium channels that acquired Ankyrin-binding capacities during chordate evolution (Hill et al., 2008) to determine the physiological properties of specific subcellular neuronal compartments. While the AnkD1 of Drosophila *ank2* had the least impact on synaptic stability phenotypes it will be interesting to identify putative binding proteins that may provide specific functional properties as absence of this domain resulted in decreased survival and locomotion of adult flies.

In contrast to Ank2-L, the deletion of individual parts of the ARD had smaller effects on synaptic targeting of Ank2-XL in the *ank2^ΔXL^* mutant background. Here, only the deletion of AnkD3 (repeats 13–18) resulted in a significant reduction of axonal and synaptic localization. However, our analysis revealed a striking dependence of Ank2-XL on the presence of the intact ARD of Ank2-L. Impairments of Ank2-L localization, including the minor effects caused by deletion of AnkD1, resulted in a severe reduction of synaptic Ank2-XL levels. This shows that Ank2-XL localizes via Ank2-L to the presynaptic terminal consistent with our prior observations (Stephan et al., 2015). Interestingly, deletions of parts of the ARD of Ank2-XL also significantly reduced synaptic Ank2-L levels indicating a critical co-dependence of Ank2-L and Ank2-XL at the NMJ. This interaction may depend on direct interactions between the two Ankyrin isoforms, potentially mediated within the ARD. However, it is equally possible that incorporation of mutated Ankyrins with altered binding properties resulted in a disruption of the larger Ankyrin-Spectrin scaffold that in turn leads to reduced targeting of the other isoform. Indeed, in vertebrate axons it has been demonstrated that different affinities of specific giant Ankyrins control the isoform-specific incorporation into selective axonal compartments like the nodes of Ranvier (Ho et al., 2014).

### Ankyrin-Dependent Control of Synaptic Stability

At a functional level we observed a strong correlation between synaptic Ank2-L level and the control of synaptic stability. At all muscle groups analyzed we observed that deletion of AnkD1 domain had the mildest impact on synaptic stability consistent with the least impairments of synaptic Ank2-L localization. Importantly, the analysis of synaptic stability at muscle 4 also revealed that AnkD3 is most critical for synaptic maintenance. Despite an almost identical reduction in Ank2-L level compared to AnkD2 and 4 the rescue construct lacking the third domain was unable to restore any synaptic stability of *ank2^ΔL^* mutant animals. Interestingly, this assay also highlighted the specificity of the individual ankyrin repeats within the ARD. The exchange of second domain sequences with analogous sequences of the first domain failed to restore both Ank2-L localization and the synaptic stability phenotype of *ank2^ΔL^* mutants. The failure to support synaptic stability is at least in part due to a failure to organize and stabilize synaptic cell adhesion molecules. Our analysis demonstrated that AnkD2 and 3 are critical for normal clustering of the NCAM homolog Fas II and of the L1 CAM homolog Nrg. This is consistent with our prior observations that Ank2-L supports synaptic organization of both Fas II and Nrg and the co-dependence of Ank2-L and these CAMs at the synapse (Pielage et al., 2008; Enneking et al., 2013). This function is evolutionary conserved as mutations in the second and third binding site of vertebrate AnkG failed to restore clustering of the L1 CAM family members Neurofascin or Nr-CAM at the AIS of hippocampal neurons (Wang et al., 2014).

### Ankyrin-XL Dependent Control of Synaptic Microtubule Organization

In contrast to the clear requirements of the ARD for presynaptic targeting of Ank2-L the same mutations only partially affected localization of Ank2-XL. Consistently, we observed only mild disruptions of the presynaptic microtubule cytoskeleton and of the synaptic bouton organization compared to the *ank2^ΔXL^* mutation (Stephan et al., 2015). The effects were most severe when deleting AnkD2 despite the fact that Ank2-XL protein levels were more compromised in *AnkD3* mutants. As the large C-terminal repeat region of Ank2-XL is essential for the interaction with microtubules (Stephan et al., 2015) these results indicate that inappropriate ARD-dependent complex assembly may influence the functional properties of Ank2-XL at the NMJ.

Together, our *in vivo* analysis of the ARD of Drosophila giant Ankyrins uncovered a structural basis for presynaptic localization and provided a genetic basis for the identification of regulatory mechanisms controlling structural synaptic plasticity via the selective Ankyrin complex assembly.

## Materials and Methods

### Fly Genetics

Flies were maintained at 25°C. Fly strains were as follows: *ank2b_p40*, *ank2^Δ*XL*^* (control rescue *ank2^Δ*XL*^*); *ank2b_p40*, *ank2*^518^ (control rescue *ank2^Δ*L*^*); *ank2^Δ*XL*^* (all Stephan et al., 2015); *ank2*^2001^ (Pielage et al., 2008); *ank2b_p40ΔAnkD, ank2^518^* (P[acman] constructs with single ankyrin repeat domain deletions for rescues in *ank2^Δ*L*^* background); *ank2b_p40ΔAnkD*, *FRT ank2^Δ*XL*^* (P[acman] constructs with single ankyrin repeat domain deletions for rescues in *ank2^Δ*XL*^* background) (all this paper).

### Generation of P[acman] Constructs

We used a 2-step *gal*K mediated recombineering protocol according to Warming et al., 2005 and instructions available at the website of the National Laboratory for Cancer Research (Frederick, United States)^1^. Insertions of a *gal*K selection cassette into the specific ankyrin repeat domains at the 5′end of the *ank2* locus (exact location given below) was performed as described in Stephan et al., 2015.

All constructs and recombineering events were verified by sequencing.

### Primers for the Generation of P[acman] Constructs

For inserting the *gal*K cassette at a specific site within the *ank2* locus primers named -gK- were used. Plasmid specific sequences required to amplify the *gal*K cassette are indicated by italic letters. To delete the galK selection cassette in the second recombineering step and to generate the final deletion or modification of *ank2* additional primers are listed. The positions are all according to the open reading frame of the 4083 aa long Ank2-L protein.

#### P[acman]-ank2-ΔAnk1

Location: deletion of aa 10 to aa 203

Ank2-ΔAnk1-gK-f

ATGAATATGAAAAACGATATTTGAACTTATGAAACTTT ATTTTCTAGGGC*CCTGTTGACAATTAATCATCGGCA*

Ank2-ΔAnk1-gK-r

ATGTTTTGGTTACCATAATGGGAAGCAATGTGCAGC GGGGTGAAGCCCGA*TCAGCACTGTCCTGCTCCTT*

Ank2-ΔAnk1-f

ATGAATATGAAAAACGATATTTGAACTTATGAAACTTT ATTTTCTAGGGCTCGGGCTTCACCCCGCTGCACATTGCT TCCCATTATGGTAACCAAAACAT

Ank2-ΔAnk1-r

ATGTTTTGGTTACCATAATGGGAAGCAATGTGCAGC GGGGTGAAGCCCGAGCCCTAGAAAATAAAGTTTCATA AGTTCAAATATCGTTTTTCATATTCAT

#### P[acman]-ank2-ΔAnk2

Location: deletion of aa 204 to aa 401

Ank2-ΔAnk2-gK-f

AATTCTTTCCCTGCTTTTCAGAATGACCATAATCCGG ACGTGACTTCCAAG*CCTGTTGACAATTAATCATCGGCA*

Ank2-ΔAnk2-gK-r

AATGTTCATGCAACCCATAAAGGCGGCCACATGGAGC GGAGTAAGTCCACT*TCAGCACTGTCCTGCTCCTT*

Ank2-ΔAnk2-f

AATTCTTTCCCTGCTTTTCAGAATGACCATAATCCGG ACGTGACTTCCAAGAGTGGACTTACTCCGCTCCATGTG GCCGCCTTTATGGGTTGCATGAACATT

Ank2-ΔAnk2-r

AATGTTCATGCAACCCATAAAGGCGGCCACATGGAG CGGAGTAAGTCCACTCTTGGAAGTCACGTCCGGATTAT GGTCATTCTGAAAAGCAGGGAAAGAATT

#### P[acman]-ank2-ΔAnk3

Location: deletion of aa 402 to aa 599

Ank2-ΔAnk3-gK-f

TGGTAGAGTTGCTCTTGCGACATGGTGCCAGCATTAG TGCCACGACAGAG*CCTGTTGACAATTAATCATCGGCA*

Ank2-ΔAnk3-gK-r

ATGTCCATCTGGTTCTTCCTTGCGGCAATATGGAGCG GGGTATGTCCATT*TCAGCACTGTCCTGCTCCTT*

Ank2-ΔAnk3-f

TGGTAGAGTTGCTCTTGCGACATGGTGCCAGCATTAG TGCCACGACAGAGAATGGACATACCCCGCTCCATATT GCCGCAAGGAAGAACCAGATGGACAT

Ank2-ΔAnk3-r

ATGTCCATCTGGTTCTTCCTTGCGGCAATATGGAGCG GGGTATGTCCATTCTCTGTCGTGGCACTAATGCTGGCAC CATGTCGCAAGAGCAACTCTACCA

#### P[acman]-ank2-ΔAnk4

Location: deletion of aa 600 to aa 797

Ank2-ΔAnk4-gK-f

GTTGCCCTGTTGCTTCTGGAGAAGGGAGCAAGTCCA CATGCCACGGCTAAG*CCTGTTGACAATTAATCATCGGCA*

Ank2-ΔAnk4-gK-r

GTGCATGGCCTCGGGAGCCACCACGCGATACTTCTCC TCGGCTTGGGAAGG*TCAGCACTGTCCTGCTCCTT*

Ank2-ΔAnk4-f

GTTGCCCTGTTGCTTCTGGAGAAGGGAGCAAGTCCA CATGCCACGGCTAAGCCTTCCCAAGCCGAGGAGAAGTAT CGCGTGGTGGCTCCCGAGGCCATGCAC

Ank2-ΔAnk4-r

GTGCATGGCCTCGGGAGCCACCACGCGATACTTCTCC TCGGCTTGGGAAGGCTTAGCCGTGGCATGTGGACTTGCT CCCTTCTCCAGAAGCAACAGGGCAAC

#### P[acman]-ank2-Ank1+1

To maintain the exon-intron structure of the *ank2* locus the *ank2 domain1+*1 construct aa 10-203 encoding for Δ*AnkD1* was amplified from the cDNA clone RH63474 encoding the open reading frame of the short Ank2 isoform (Ank2-S, Pielage et al., 2008) using the following primer:

Location: Deletion/Modification of aa 203 to aa 401

Ank2-ΔAnk1+1-PCR-f

CACGCAGGAAGGAAGTGTTTCCATCCTTGGAAGTCAC GTCCGGATTATGGTCATTCTGAAAAGCAGGGAAAGAATT AAAG

Ank2-ΔAnk1+1-PCR-r

AATGTTCATGCAACCCATAAAGGCGGCCACATGGAGC GGAGTAAGTCCACTCTTGGAAGTCACGTCCGGATTATG

### Generation of Transgenic Flies

We used the phiC31-integration method to generate transgenic flies (Bischof et al., 2007). All P[acman]-based constructs were integrated into the attP40 landing site to ensure identical genetic backgrounds. As previously described (Stephan et al., 2015), we transferred final P[acman] constructs to *Escherichia coli* EPI300 cells and induced high-copy amplification to prepare DNA for injection (Venken et al., 2006, 2009). Using the NucleoBond BAC 100 kit (Machery-Nagel) DNA was prepared following the manufacturer’s instructions and resuspended in 10 mM Tris (pH 8.0) buffer. Using restriction enzymes DNA fingerprints were generated and analyzed to control DNA integrity. Before injection, DNA was diluted in 10 mM Tris (pH 8.0) to a final concentration of 80 ng/μl.

### Immunohistochemistry

Second and third instar wandering Larvae were dissected in ice-cold dissecting saline (HL3) at room temperature and fixed for 5 min in Bouin’s fixative (Sigma Aldrich). Third instar larvae were incubated with primary antibodies for 2 h at room temperature in phosphate buffered saline containing 0.1% Triton X-100. The following primary antibodies were used: mouse anti-Brp (nc82, 1:250), mouse anti-Futsch (22C10, 1:500), mouse anti-Fas II (1D4, 1:60) (all Developmental Studies Hybridoma Bank, Iowa); rabbit anti-GluRIIc (1:3000; Pielage et al., 2011); rabbit anti-Ank2-XL (1:2000; Koch et al., 2008); rat anti-Ank2-L (1:40; Enneking et al., 2013). For second instar larvae primary antibodies were applied overnight at 4°C. Secondary antibodies (Alexa-coupled) were used at 1:1000 for 1 h at room temperature. Conjugated anti-HRP (Jackson Immunoresearch Laboratories) were used at 1:500 together with the secondary antibody for 1 h at room temperature. Larvae were mounted on slides using Prolong Gold antifade reagent.

Images were acquired at room temperature using a Zeiss LSM 700 confocal microscope with a 63 × 1.4 NA oil objective using Zen 2010 software (Zeiss). Calibrated confocal images were used for all quantitative Intensity, diameter and FasII cluster per area measurements and analyzed using FIJI/IMAGEJ and Microsoft Excel.

### Analysis of Synaptic Stability

Synaptic retractions were quantified using antibodies against the presynaptic active zone marker Brp and against the GluRIIc subunit of the postsynaptic glutamate receptors. Retraction frequency indicates the percentage of NMJs per animal showing GluRIIc staining no longer opposed by Brp in segments A3–A5 on muscles 6/7, 4, or 1. Synaptic retraction severity indicates the number of postsynaptic bouton profiles per NMJ that were not opposed by Brp. One to two retracted synaptic boutons were considered a small retraction, three to six a medium retraction and more than six or a complete elimination was considered a large retraction.

### Analysis of CAM Clustering

Images for Fas II and Nrg measurements were acquired on segments A2-A3 of muscle 4. Quantifications were performed using a custom-made macro in FIJI/IMAGEJ (available on request). Fas II cluster per NMJ area were calculated using HRP as a marker for total NMJ area. NMJ area represents the entire NMJ from proximal to distal boutons. Fas II clusters were counted within this area to quantify Fas II cluster per NMJ. Cluster frequency indicates the percentage of NMJs showing Fas II clusters. Fas II cluster number was normalized to 100 μm^2^ using the NMJ area measured before to calculate the cluster density.

### Analysis of Futsch Organization

Futsch organization was scored as aberrant when NMJs contained irregular thinnings or thickenings of the core filaments. All phenotypes were quantified on muscle 4 NMJs in segments A2–A5 and are presented as percentage of affected NMJs. For Futsch diameter measurements images were taken on muscle 4 in segments A2 and A3. The thickness of Futsch filaments was measured every 2 μm within the region of the largest synaptic bouton to determine the largest diameter. Using the same technique and the profile command of FIJI/IMAGEJ Futsch distribution within boutons was determined.

### Intensity Measurements of Ank2 Level

For Ank2-L and Ank2-XL intensity measurements, larvae of all genotypes including controls were stained together in one single tube. Images were taken on muscle 4 in segments A2 or A3. Image settings were set for control larvae and then applied to all genotypes. All genotypes of a given dataset were imaged within a single imaging session. Using a custom-made FIJI macro (available on request) maximum intensity projections were generated and the HRP signal was used to mask the area for Ank2-L and Ank2-XL intensity measurements. For axonal values, the largest area of the axon was selected. For NMJ measurements, the entire NMJ was selected. For distal boutons measurements the two most distal boutons were selected. For proximal boutons measurements the two most proximal boutons after NMJ innervation were selected. For each measurement background intensity was measured and subtracted using (integrated density − (area × mean background)). Genotypes were normalized to controls.

### Statistical Analysis

Statistical analysis was performed using GraphPad (Prism). Normal distribution was determined by D’agostino-Pearson omnibus and Shapiro–Wilk normality tests. For normally distributed data a one–way ANOVA with Dunnett’s multiple comparison test correction was used. For non-normally distributed data a Kruskal–Wallis test with Dunn’s multiple comparison test correction was used. *p* < 0.05 was accepted as statistically significant (^*^p < 0.05, ^∗∗^*p* < 0.01, ^∗∗∗^*p* < 0.001). In all graphs data are represented as mean ± SEM.

## Data Availability

All datasets generated for this study are included in the manuscript and/or the Supplementary Files.

## Author Contributions

TW performed all experiments. RS designed the P[acman] vectors and generated the transgenic flies. RS together with EM cloned the constructs. TW and JP designed the experiments, analyzed the data, and wrote the manuscript.

## Conflict of Interest Statement

The authors declare that the research was conducted in the absence of any commercial or financial relationships that could be construed as a potential conflict of interest.

## Footnotes

1 https://ncifrederick.cancer.gov/Research/Brb/#/recombineeringInformation
