# Supplementary material for: The Ankyrin Repeat Domain Controls Presynaptic Localization of Drosophila Ankyrin2 and Is Essential for Synaptic Stability
Source: Front Cell Dev Biol. 2019 Aug 14;7:148. doi: 10.3389/fcell.2019.00148 (PMC6703079; doi:10.3389/fcell.2019.00148)
Supplement: Supplementary file 1 [file Data_Sheet_1.PDF]

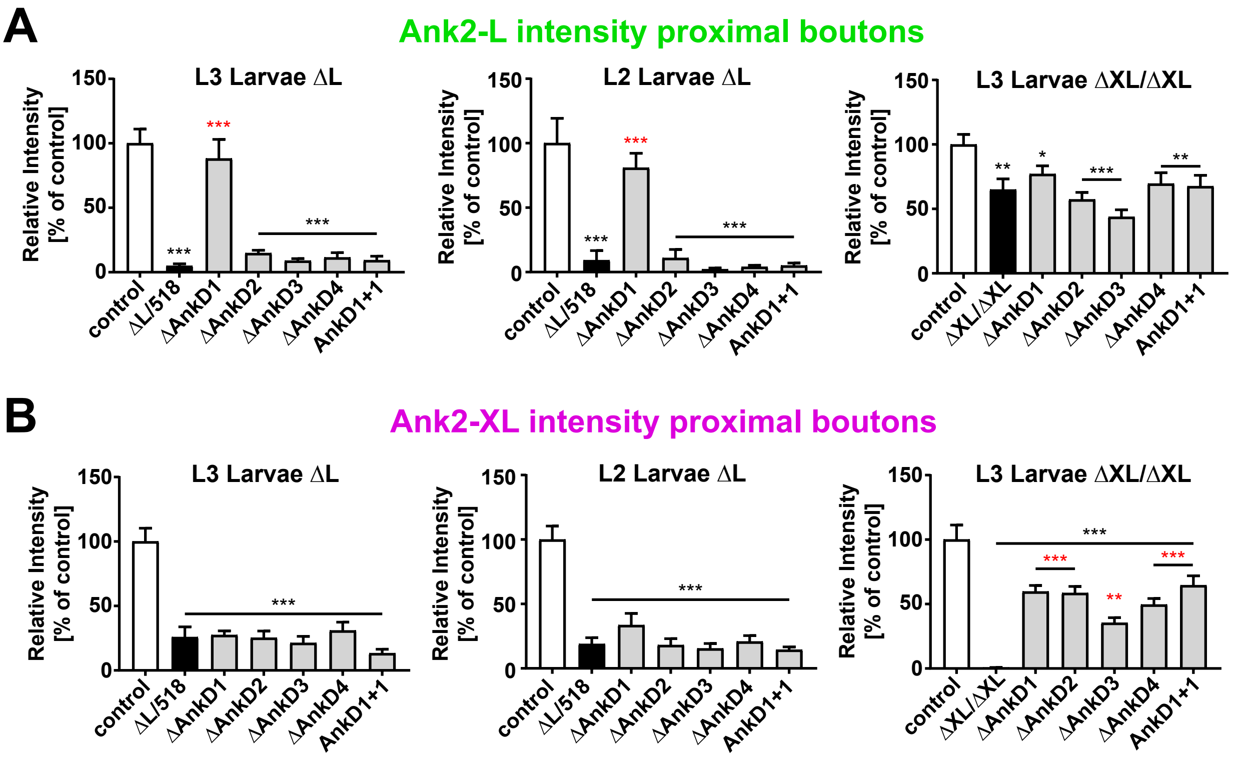


**Figure S1: Analysis of Ank2-L and Ank2-XL localization in proximal synaptic boutons**
(A) Quantification of Ank2-L level in proximal synaptic boutons in third (L3) and second (L2) instar larvae in *ank2*$^{\Delta L}$ mutant animals and in third instar larvae in the *ank2*$^{\Delta XL}$ mutant animals (n = 11–12 muscle 4 NMJs, 3 animals).
(B) Quantification of Ank2-XL level in proximal synaptic boutons in third (L3) and second (L2) instar larvae in *ank2*$^{\Delta L}$ mutant animals and in third instar larvae in the *ank2*$^{\Delta XL}$ mutant animals (n = 11–12 muscle 4 NMJs, 3 animals).
Error bars indicate SEM; *p < 0.05, **p < 0.01, ***p < 0.001 (ANOVA); black asterisks represent comparison to controls; red asterisks represent comparison to *ank2*$^{\Delta L}$ and *ank2*$^{\Delta XL}$ mutants.
