# Supplementary material for: The Ankyrin Repeat Domain Controls Presynaptic Localization of Drosophila Ankyrin2 and Is Essential for Synaptic Stability
Source: Front Cell Dev Biol. 2019 Aug 14;7:148. doi: 10.3389/fcell.2019.00148 (PMC6703079; doi:10.3389/fcell.2019.00148)
Supplement: Supplementary file 2 [file Data_Sheet_2.PDF]

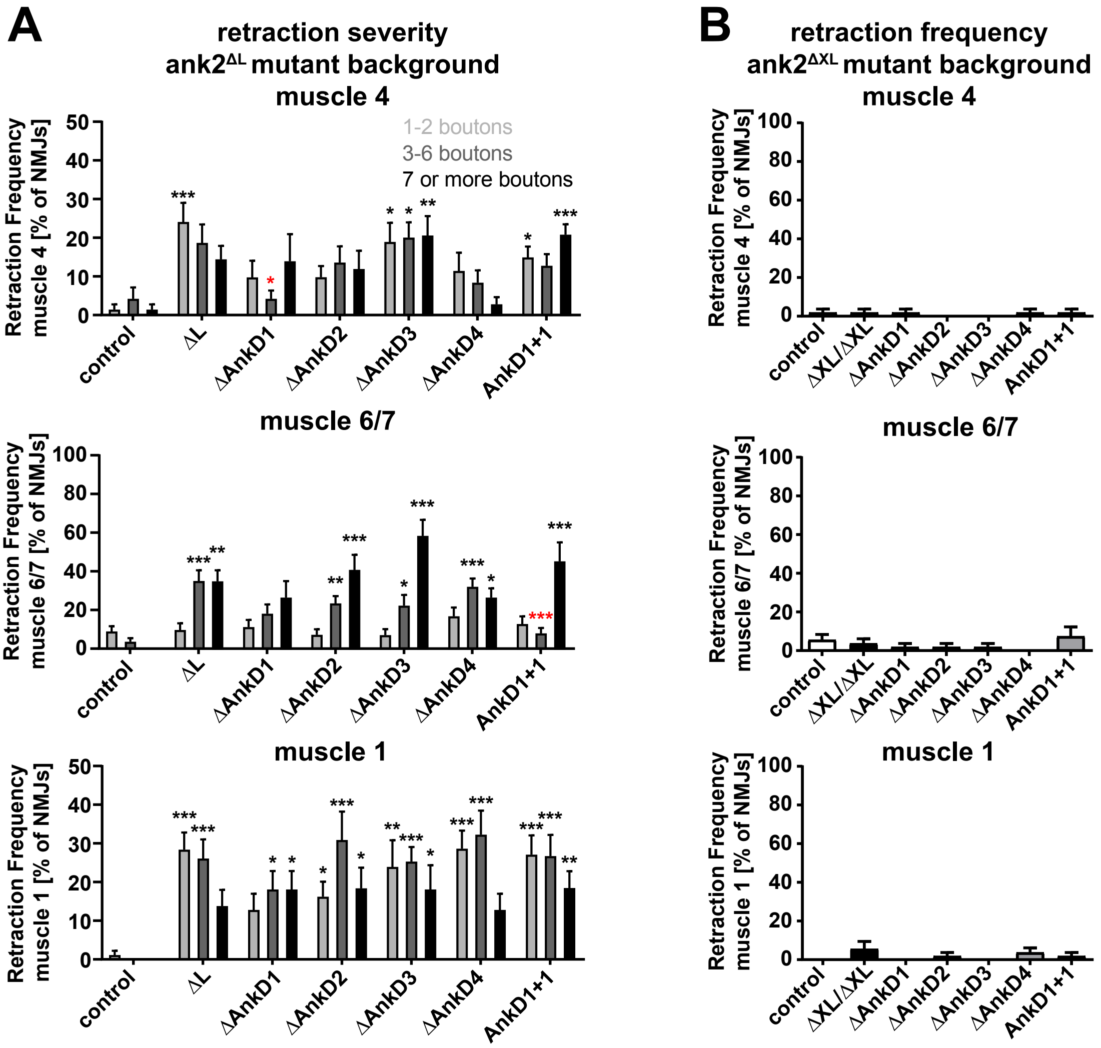


**Figure S2: Analysis of synaptic retraction severity**

(A) Analysis of the severity of synaptic retractions on muscle 4, muscle 6/7 and muscle 1 in controls, *ank2*$^{\Delta L}$ and ARD deletion mutants in the *ank2*$^{\Delta L}$ mutant background (n = 72–111 muscle 6/7 NMJs, 12–19 animals; n = 66–101 muscle 4 NMJs, 11–19 animals; n = 71–104 muscle 1 NMJs; 12–19 animals).

(B) Analysis of the frequency of synaptic retractions on muscle 4, muscle 6/7 and muscle 1 in controls, *ank2*$^{\Delta XL}$ and ARD deletion mutants in the *ank2*$^{\Delta XL}$ mutant background (n = 54 muscle 6/7 NMJs, 9 animals; n = 54 muscle 4 NMJs, 9 animals; n = 54 muscle 1 NMJs, 9 animals).

Error bars indicate SEM; *p < 0.05, **p < 0.01, ***p < 0.001 (ANOVA); black asterisks represent comparisons to controls; red asterisks represent comparisons to Ank2-L mutants.
