# Supplementary material for: The Ankyrin Repeat Domain Controls Presynaptic Localization of Drosophila Ankyrin2 and Is Essential for Synaptic Stability
Source: Front Cell Dev Biol. 2019 Aug 14;7:148. doi: 10.3389/fcell.2019.00148 (PMC6703079; doi:10.3389/fcell.2019.00148)
Supplement: Supplementary file 3 [file Data_Sheet_3.pdf]

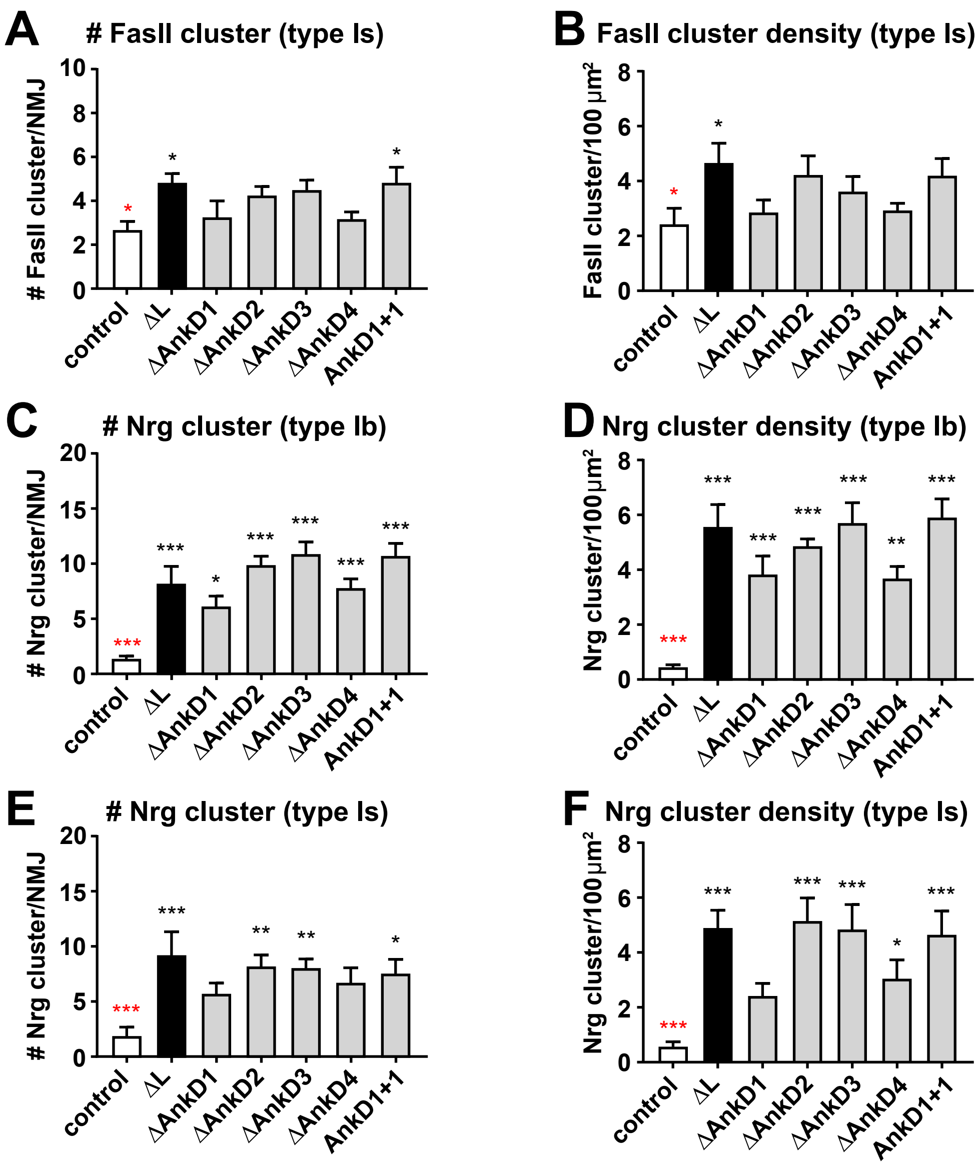

Figure S3: Analysis of FasII and Nrg organization at type Is/Ib muscle 4 NMJs.

(A-B) Quantification of FasII cluster number (A) and density (B) at type Is NMJs (n = 12 muscle 4 NMJs, 3 animals).

(C-D) Quantification of Nrg cluster number (C) and density (D) at type Ib NMJs (n = 12 muscle 4 NMJs, 3 animals).

(E-F) Quantification of Nrg cluster number (E) and density (F) at type Is NMJs (n = 12 muscle 4 NMJs, 3 animals). Error bars indicate SEM; *$p < 0.05$, **$p < 0.01$, ***$p < 0.001$ (ANOVA); black asterisks represent comparison to controls; red asterisks represent comparison to *ank2*$^{\Delta L}$.
