# Supplementary material for: The Ankyrin Repeat Domain Controls Presynaptic Localization of Drosophila Ankyrin2 and Is Essential for Synaptic Stability
Source: Front Cell Dev Biol. 2019 Aug 14;7:148. doi: 10.3389/fcell.2019.00148 (PMC6703079; doi:10.3389/fcell.2019.00148)
Supplement: Supplementary file 4 [file Data_Sheet_4.PDF]

Ank2-L/Ank2-XL	ARD 1	ARD 2	ARD 3	ARD 4
Ank2 localization (in ΔL mutants)	+++<br>++	+++++<br>+++	+++++<br>++++	+++++<br>+++
Ank2 localization (in ΔXL mutants)	+++<br>+	++++<br>+	+++++<br>++++	+++<br>+++
Futsch organization (in ΔXL mutants)	+	++	+	+
FasII organization (in ΔL mutants)	++	++++	++++	+++
Nrg organization (in ΔL mutants)	+++	++++	++++	+++
Synaptic stability (in ΔL mutants)	+	++++	++++	++

Figure S4: Summary of the synaptic requirements of the different ARDs.

The table shows the requirements of the different ARD subunits for Ank2 isoform localization and for functional synaptic parameters. The number of crosses indicates the importance for the different processes with five crosses indicating highest requirements. The first two rows show the differential requirements for the localization of Ank2-L (green) and Ank2-XL (magenta) in the isoform specific mutant backgrounds. The last 4 rows summarize the importance of the different ARD subunits for Futsch, FasII and Nrg organization and for the maintenance of synaptic stability.
